# Differential Responses of CO_2_ Assimilation, Carbohydrate Allocation and Gene Expression to NaCl Stress in Perennial Ryegrass with Different Salt Tolerance

**DOI:** 10.1371/journal.pone.0066090

**Published:** 2013-06-14

**Authors:** Tao Hu, Longxing Hu, Xunzhong Zhang, Pingping Zhang, Zhuangjun Zhao, Jinmin Fu

**Affiliations:** 1 Key Laboratory of Plant Germplasm Enhancement and Specialty Agriculture, Wuhan Botanical Garden, The Chinese Academy of Science, Wuhan, China; 2 Virginia Polytechnic Institute and State University, Blacksburg, United States of America; Arizona State University, United States of America

## Abstract

Little is known about the effects of NaCl stress on perennial ryegrass (*Lolium perenne* L.) photosynthesis and carbohydrate flux. The objective of this study was to understand the carbohydrate metabolism and identify the gene expression affected by salinity stress. Seventy-four days old seedlings of two perennial ryegrass accessions (salt-sensitive ‘PI 538976’ and salt-tolerant ‘Overdrive’) were subjected to three levels of salinity stress for 5 days. Turf quality in all tissues (leaves, stems and roots) of both grass accessions negatively and significantly correlated with GFS (Glu+Fru+Suc) content, except for ‘Overdrive’ stems. Relative growth rate (RGR) in leaves negatively and significantly correlated with GFS content in ‘Overdrive’ (*P*<0.01) and ‘PI 538976’ (*P*<0.05) under salt stress. ‘Overdrive’ had higher CO_2_ assimilation and F_v_/F_m_ than ‘PI 538976’. Intercellular CO_2_ concentration, however, was higher in ‘PI 538976’ treated with 400 mM NaCl relative to that with 200 mM NaCl. GFS content negatively and significantly correlated with RGR in ‘Overdrive’ and ‘PI 538976’ leaves and in ‘PI 538976’ stems and roots under salt stress. In leaves, carbohydrate allocation negatively and significantly correlated with RGR (r^2^ = 0.83, *P*<0.01) and turf quality (r^2^ = 0.88, *P*<0.01) in salt-tolerant ‘Overdrive’, however, the opposite trend for salt-sensitive ‘PI 538976’ (r^2^ = 0.71, *P*<0.05 for RGR; r^2^ = 0.62, *P*>0.05 for turf quality). A greater up-regulation in the expression of *SPS*, *SS*, *SI*, *6-SFT* gene was observed in ‘Overdrive’ than ‘PI 538976’. A higher level of *SPS* and *SS* expression in leaves was found in ‘PI 538976’ relative to ‘Overdrive’. Accumulation of hexoses in roots, stems and leaves can induce a feedback repression to photosynthesis in salt-stressed perennial ryegrass and the salt tolerance may be changed with the carbohydrate allocation in leaves and stems.

## Introduction

Salinity is a severe and increasing threat limiting plant growth and crop yields worldwide [1], [2]. There are approximately 6% of the world’s total land area and 50% irrigated lands being severely affected by salinity [3], [4].

Previous studies have shown that salt stress affects CO_2_ assimilation in many plants including cotton (*Gossypium hirsutum* L.) [5], [6], bean (*Phaseolus vulgaris* L.) [7], [8], bell pepper (*Capsicum annuum* L.) [9], celery (*Apium graveolens* L.) [10], spinach (*Spinacia oleracea* L.) [11], rice (*Oryza sativa* L.) [12], sea barleygrass (*Hordeum marinum* Huds.), cultivated barley (*Hordeum vulgare* L.) [13] and tomato (*Solanum lycopersicum*) [14]. Meloni et al. [6] observed a lower level of CO_2_ assimilation and stomatal conductance with increasing NaCl concentration in cotton (*Gossypium hirsutum* L.) and concluded that stomatal aperture limited leaf photosynthetic capacity in the NaCl-treated plants. Similar results have been obtained for cotton and bean (*Phaseolus vulgaris* L.) [5], spinach [11], rice [12], and sea barleygrass and cultivated barley [13]. Other studies, however, proved that non-stomatal factors play a key role in the response of leaf CO_2_ assimilation to salinity environment, such as ion toxicity [9], PSII activity and photophosphorylation activity [10], enzyme activity [15] and salt tolerant gene expression [14]. Photochemistry efficiency (F_v_/F_m_) as an important parameter has been widely used as an indicator of photoinhibition of photosynthesis in higher plants to environmental stresses [16], [17]. Therefore, the mechanisms by which salt stress leads to a decrease in CO_2_ assimilation are still not yet clearly understood.

Sugars are primary products of photosynthesis in higher plants [18]. Soluble sugars (i.e. sucrose, glucose and fructose) are highly sensitive to environmental stresses and the major forms of carbohydrates. Sugars not only provide energy and solutes for osmotic adjustment, but also modulate expression of multiple genes as regulatory messenger through the sugar sensing and signaling network in many metabolic processes [19]–[22]. Soluble sugar is a very dynamic cycling process of degradation and synthesis in carbohydrates metabolism [23], [24]. Biotic and abiotic stresses alter sugar concentration and metabolic ﬂux [25], [26]. It is important to determine soluble sugar and sugar ﬂux for understanding feed-forward and feed-back control of photosynthesis in plants *response* to salinity stress.

Plants can increase a regulatory level of sugar signal molecules by reprogramming the expression of endogenous genes [27]. Lower levels of transcripts of the genes sucrose phosphate synthase (*SPS*), sucrose synthase (*SS*) and cell wall sucrose invertase (*SI*) were maintained in the WT sugar beet (*Beta vulgaris* L.) compared with the transgenic lines at 300 mM NaCl [28]. It was reported that sucrose: sucrose 6-fructosyltransferase (*6-SFT*) involved a signal regulating in grass fructan biosynthesis [29]. It is important to elucidate the expression of candidate genes involved in the regulation pathways in carbohydrate metabolism for understanding molecular adaptations of plants to salinity stress.

Perennial ryegrass (*Lolium perenne* L.) is an important forage grass and cool-season turfgrass species and cultivated in United States, Europe, Japan, Australia and New Zealand because of its rapid establishment rate and good wear tolerance [30]. Although the effects of salinity stress on germination and growth [31], [32], chlorophyll content [28], transgenic effect [33] and the antioxidant system [34] of perennial ryegrass have been investigated, there is limited information on photosynthesis and carbohydrate allocation of perennial ryegrass in response to salt stress. The objective of this study were: (1) to investigate the difference in mechanisms by which salt stress lead to a decrease in CO_2_ assimilation and (2) the differential responses of carbohydrate allocation and gene expression in perennial ryegrass accessions contrasting in salt tolerance.

## Materials and Methods

### Plant Materials and Growth Conditions

The seeds of two perennial ryegrass accessions, salt sensitive ‘PI 538976’ and salt tolerant ‘Overdrive’ were used for this study. The seeds were planted in plastic cups (10 cm in diameter and 15 cm deep) filled with sand and covered with a 0.5-cm layer of sand. The bottom of each cup was drilled (5 mm in diameter), to allow drainage of excess water and soil aeration. When the plants were 8 cm tall, they were mowed at a 5 cm height. The grass was mowed at the same height three times weekly thereafter. In addition, the seedings were irrigated daily and fertilized twice weekly with half-strength Hoagland’s solution [35]. After a 2-month of growth period, the plants were rinsed thoroughly using distill water and transferred into 300 ml erlenmeyer flasks filled with approximately 290 ml half-strength Hoagland’s solution. The flasks were wrapped with aluminum foil to prevent potential growth of algae and the bottlenecks were closed with a proper amount of absorbent paper. All flasks were kept in a greenhouse with daily temperature of 21/18°C (day/night), photosynthetic active radiation (PAR) at 300 µmol m^−2^ s^−1^, and a 14 h photoperiod. The transpiration rate of each flask was calculated based on the difference in weight of plant-flask system at the beginning of the study. Plants with similar transpiration rate were selected for each replicate of the NaCl treatments. Plants were allowed to grow in the above-mentioned conditions for 2 weeks before the NaCl treatments were initiated.

### Treatments and Experiment Design

After 2-week period of pre-adaptation, perennial ryegrass was subjected to three salinity levels (0, 200 and 400 mM NaCl) in each flask by adding NaCl to the half-strength Hoagland nutrition. Each flask contained 0.1 µmol magnesium oxide for providing plants additional oxygen. The flasks were sealed with plasticene covered with preservative film and wrapped with silicon sealant to prevent escape of water or chemicals. Each treatment maintained the final concentration for 5 days. At the end of the experiment, the roots, stems and leaves were harvested separately. Treatments and grass accessions were arranged in a randomized complete block design with four replicates.

### Measurements

Vertical canopy height of each grass before and after treatment was measured. Average relative growth rate (RGR) was calculated by Eq 1, where H_0_ and H_t_ denote initial and final height between two adjacent marks, respectively, and 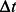 the duration of the experiment (5 d).
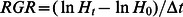
(1)


As the second parameter for evaluating growth status, turf quality was rated visually based on turfgrass color (percentage green leaves), plant density and degree of leaf wilting on a scale of 0 to 9 with 0 score indicating grass being withered and yellow, thin and dead, and 9 score indicating the grass being green, dense, uniform and 6 being the minimum acceptable level [36].

The transpiration rate of plants is a significant physiological index reflecting toxic effects under abiotic stress [37]. The plant-flask system was weighed every day for determining transpiration rate (i.e. water loss). The relative transpiration was normalized with respect to the initial and non-contaminated transpiration. The mean normalized relative transpiration (NRT) was calculated by Eq 2:
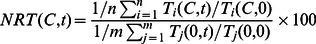
(2)where *C* is the concentration (mg L^−1^), *T* represents the absolute transpiration of the grass (g d^−1^), *t* is time period (0–1, 1–2 d, etc.), *i* is the replicate 1, 2,*…*, *n* and *j* is control 1, 2,*…*, *m*. The NRT of controls is always set at 100%. The NRT <100% indicates a inhibition of grass’s transpiration, the NRT >100% stimulation.

CO_2_ assimilation (P_n_), stomatal conductance (g_s_), and intercellular CO_2_ concentration (C_i_) were measured with 4 fully expanded leaves (second from the top) from each pot. Measurements were made with a portable Li-6400/xt gas-exchange system (Li-6400/xt, LICOR, Inc, Lincoln, NB) at 500 µmol s^−1^ ﬂow rate (leaf temperature of 20±0.4°C, 60±5% relative humidity) under a controlled light intensity of 600 µmol m^−2^ s^−1^.

To determine leaf photochemical efficiency (maximum quantum efficiency of PSII), the sections of intact leaves were darkened for 30 min with leaf clips. The ratio of variable to maximum fluorescence of chlorophyll (F_v_/F_m_) was measured with Handy Plant Efficiency Analyser (PAM-2500, Hansatech Instruments Limited, Norfolk, UK) according to Gutiérrez et al. [38].

Soluble sugars in roots, stems and leaves of perennial ryegrass were extracted and assayed according to Morvan-Bertrand et al. [39]. Dry ground tissues (100 mg) were extracted in 2 ml of 92% ethanol for 10 min under intensive oscillation at 25°C. The sample was centrifuged at 20820 g for 10 min and the residue was re-extracted a further two times with 2 ml of 92% ethanol. The three supernatants were pooled and evaporated to dryness under vacuum. The ethanol and aqueous extracts were dissolved in 0.3 ml water and filtered through a 0.45-µm nylon membrane before analysis by HPLC. The soluble sugars in aliquots of carbohydrate extracts were determined using a Waters HPLC system consisting of a model 717 autosampler, model 515 pump, and Water 2410 refractive index detector (Waters Corp., MA, USA). The separation was completed on a crest amino column (4.6×250 mm, 5 µm, Boston Analytics, Inc. USA). The mobile phase consisted of acetonitrile/water (50/50, v/v) with isocratical elution at a flow rate of 1.0 ml min^−1^. The temperature of column oven was set at 40°C.

### Analysis of Gene Expression

Total RNA was extracted from the leaf tissues using Trizol reagent according to the manufacturer’s instructions. In order to remove the genomic DNA contamination, RNA samples were treated with RNase-free DNaseI. The concentration and quality of RNA preparations were determined by measuring the absorbance at 260 nm and 280 nm in a spectrophotometry (UV-2600, UNICO Instruments Co., Ltd., Shanghai, China) and checked by running a gel electrophoresis in 1.5% agarose gels with 1 µl RNA ( = 0.5 µg µl^−1^).

Reverse transcription of the purified RNA was performed at 42°C for 60 min in 20 µl reaction mixture including 2 µg RNA, 1 µl oligo (dT)_18_ primer, 5×reaction buffer, 1 µmol dNTPs, 20 units of RNase inhibitor and 200 units of M-MuLV Reverse Transcriptase, using the first strand cDNA synthesis kit (Fermentas, Canada). The first cDNA template was diluted 6-fold and kept at −20°C for RT-PCR amplification analysis. Primer sequences used to amplify the genes of interest are listed in Table 1. *YT521-B* gene was used as the reference gene [82]. The PCR reactions were performed in a Biometra Uno II thermal cycler consisting of an initial denaturing step at 95°C for 3 min, followed by 45 cycles of amplifications as follows: 10 s at 94°C, 20 s annealing for different primer at 55–58°C, and 20 s at 72°C, with a final elongation of 5 min at 72°C. Amplified products were visualized by 1.6% (w/v) agarose gel and stained in ethidium bromide (0.5 µg/ml) after running at 100 V for 40 min in 1×TE buffer (10 mM Tris, 1 mM EDTA). The digital images of the gels were visualized and photographed by the Gel Doc XR system (Bio-rad, USA).

**Table 1:** Primer sequences for RT-PCR amplification analysis in perennial ryegrass.

Gene	Primers Sequences(5′–3′)		Tm (°C)	Reference
*SPS*	F	GGCACGAGGCTCTCTGTG	55	Liu et al., 2008
	R	CCGACTCCATGAACGATG		
*SS*	F	CCGTTCATTCTGTTTTTACTAC	55	Liu et al., 2008
	R	CAGGAATGGTGGTCAGGAAC		
*SI*	F	CCTATTTTACCAGTACAATCCC	58	Liu et al., 2008
	R	CCAACCAAG CAAAATCCTC		
*6-SFT*	F	GACCGCCTGGTACGACGAGT	58	Wei and Chatterton, 2001
	R	TCCATGCTCGCCTTCAACAC		
*YT521-B*	F	TGT AGC TTG ATC GCA TAC CC	55	Lee et al., 2010
	R	ACT CCC TGG TAG CCA CCT T		

### Statistical Analysis

The data were subjected to two-way analysis of variance. Mean (±SE) separations of results for each sample and plot were performed with least significant difference (LSD) test at 5% probability level using the SAS statistical software package (SAS 9.0 for windows, SAS Institute Inc., Cary, NC). All results within the experiment were expressed as mean of four replicates.

## Results

### Growth Rate and Transpiration

**Figure 1:**
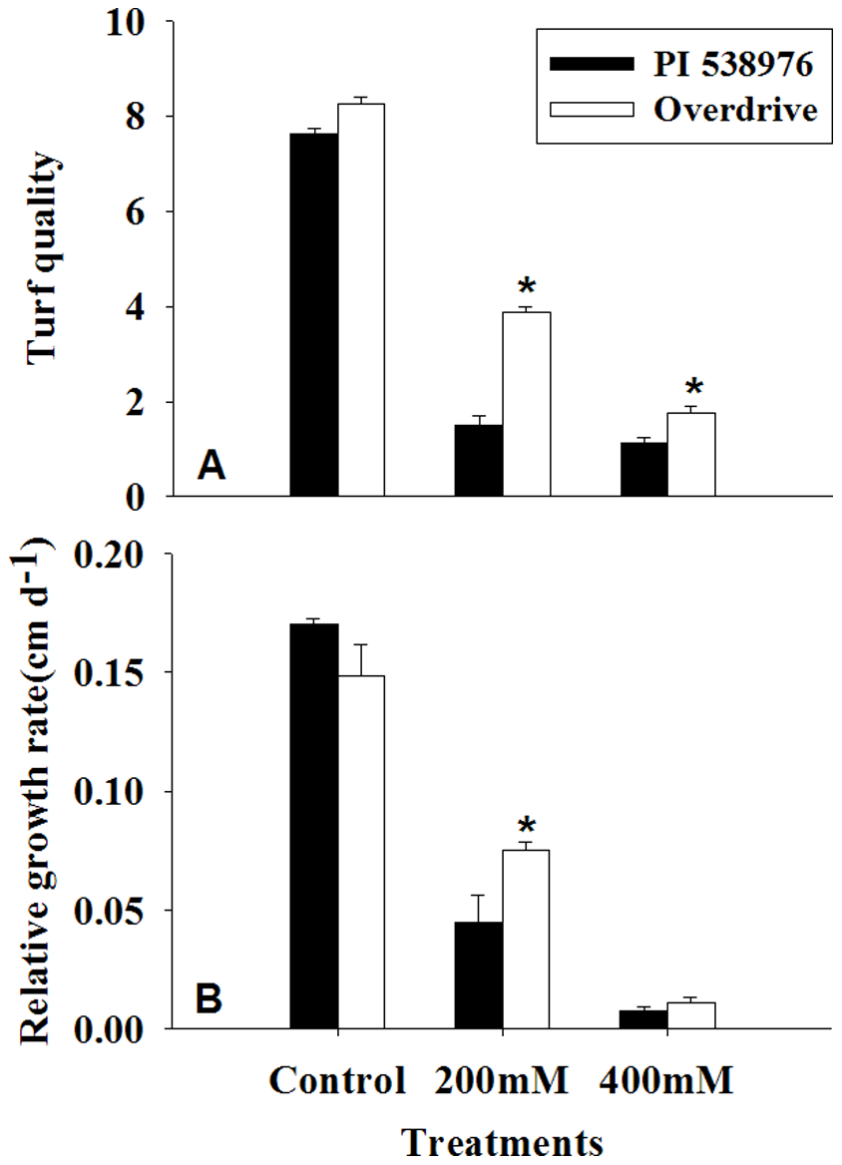
Effects of salt stress on turf quality (A) and relative growth rate (B) in ‘PI 538976’ and ‘Overdrive’. Vertical bars represent means ± standard errors (n = 4) based on least significant difference (LSD) test (*P* ≤ 0.05). Asterisk symbols indicate significant differences between ‘PI 538976’ and ‘Overdrive’ (*P* ≤ 0.05).

**Figure 2:**
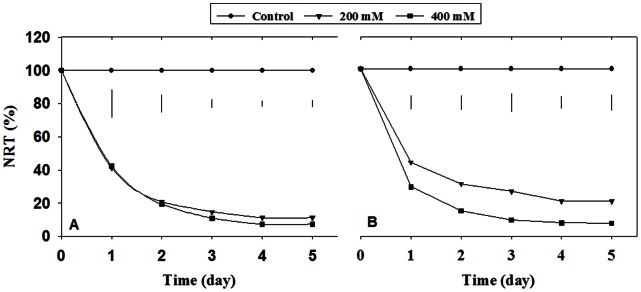
Normalized relative transpiration (NRT) of two perennial ryegrass accessions ‘PI 538976’ (A) and ‘Overdrive’ (B) in response to different levels of NaCl stress. Vertical bars on the top of each figure were least significant difference (LSD) test (*P* ≤ 0.05).

Salt stress reduced turf quality and RGR in two perennial ryegrass accessions (Fig. 1), with a larger extent in salt-sensitive ‘PI 538976’ than salt-tolerant ‘Overdrive’. Turf quality was better in ‘Overdrive’ than ‘PI 538976’ subjected to 200 mM and 400 mM NaCl (Fig. 1A). The RGR was 73.8% and 95.4% lower in ‘PI 538976’ and 49.2% and 92.7% in ‘Overdrive’ subjected to 200 mM and 400 mM NaCl, respectively, when compared to the control (Fig. 1B). Salt stress resulted in a lower level of NRT regardless of NaCl level in two perennial ryegrass accessions during whole experimental period (Fig. 2). The salinity stress (200 mM NaCl) caused a greater reduction in NRT in ‘PI538976’ relative to ‘Overdrive’. The salinity stress (200 mM NaCl) reduced NRT by 79.3% in ‘PI 538976’ and 68.6% in ‘Overdrive’ when compared to control as measured at 2 d after treatment (DAT) (Fig. 2A, B).

### Leaf Photosynthetic Traits

**Figure 3:**
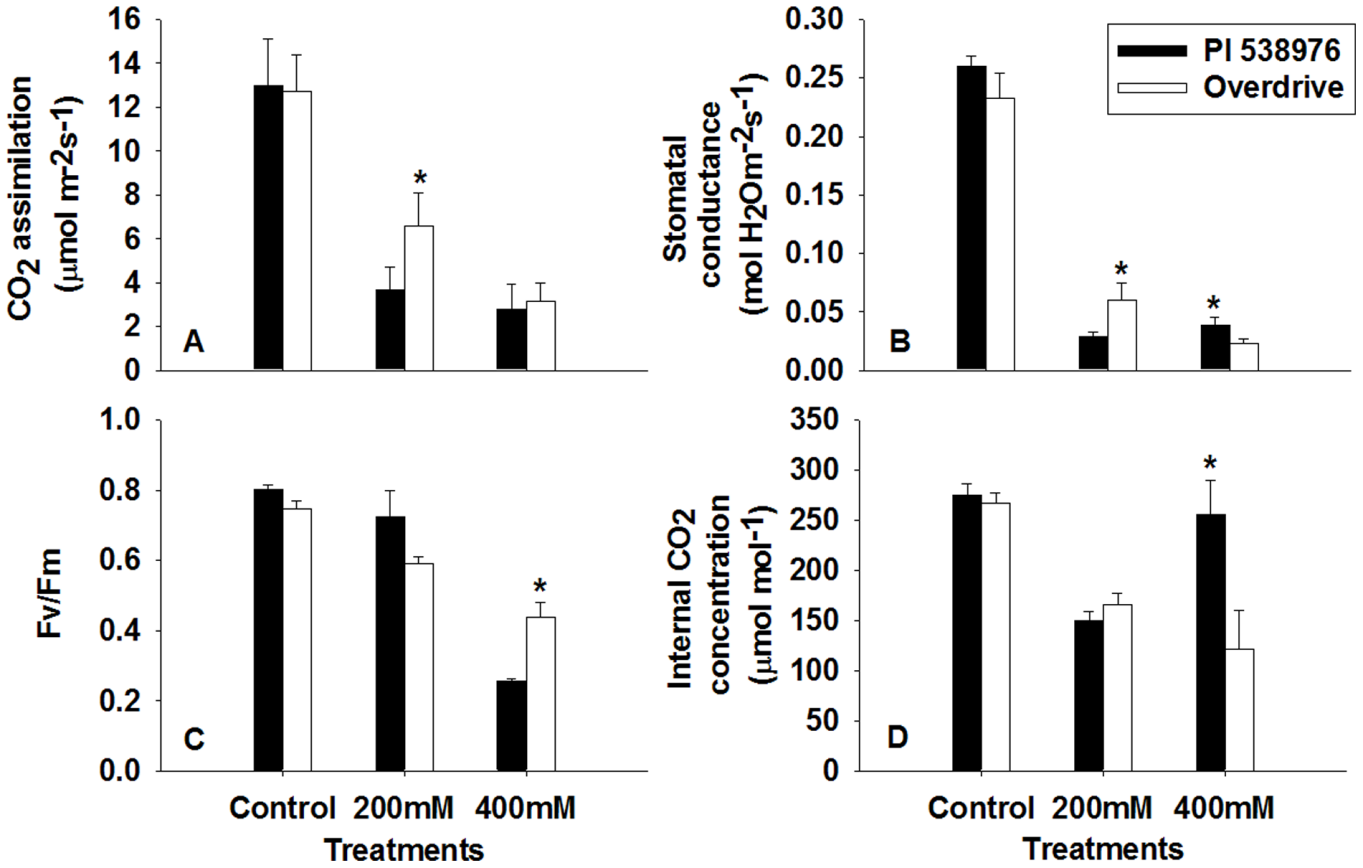
Effects of salt stress on CO_2_ assimilation (P_n_), stomatal conductance (g_s_), internal CO_2_ concentration (C_i_) and photochemical efficiency (F_v_/F_m_) in two perennial ryegrass accessions ‘PI 538976’ and ‘Overdrive’. Vertical bars represent means ± standard errors (n = 4) based on least significant difference (LSD) test (*P* ≤ 0.05). Asterisk symbols indicate significant differences between ‘PI 538976’ and ‘Overdrive’ (*P* ≤ 0.05).

Salinity stress reduced CO_2_ assimilation (P_n_), stomatal conductance (g_s_), and intercellular CO_2_ concentration (C_i_) for both perennial ryegrass accessions (Fig. 3). P_n_ was decreased by 71.7% and 78.5% for ‘PI 538976’ subjected to 200 mM and 400 mM NaCl, respectively, when compared to the control at 5 DAT. The NaCl at 200 mM and 400 mM reduced photosynthetic rate by 48.3% and 75.3%, respectively, in ‘Overdrive’ when compared to the control (Fig. 3A). The NaCl at 200 mM and 400 mM reduced leaf g_s_ by 11.1% and 14.8%, respectively, relative to the control for ‘PI 538976’ at 5 DAT. In ‘Overdrive’, the salinity stress at 200 mM and 400 mM NaCl reduced leaf g_s_ by 25.7% and 9.8%, respectively, relative to the control at 5 DAT (Fig. 3B). Leaf C_i_ was the lowest for ‘PI 538976’ treated with 200 mM NaCl, but increased by 70.2% for this accession treated with 400 mM NaCl at 5 DAT. ‘PI 538976’ had a higher level of g_s_ and C_i_ than ‘Overdrive’ when subjected to 400 mM NaCl at 5 DAT (Fig. 3D). The untreated plants maintained above a 0.75 level of leaf F_v_/F_m_ during whole experimental period for both perennial ryegrass accessions. The F_v_/F_m_ declined in both accessions as NaCl concentration increased. When exposed to 400 mM NaCl, ‘Overdrive’ had a greater F_v_/F_m_ level than ‘PI 538976’ at the end of the experiment (Fig. 3C).

### Soluble Sugar

**Figure 4:**
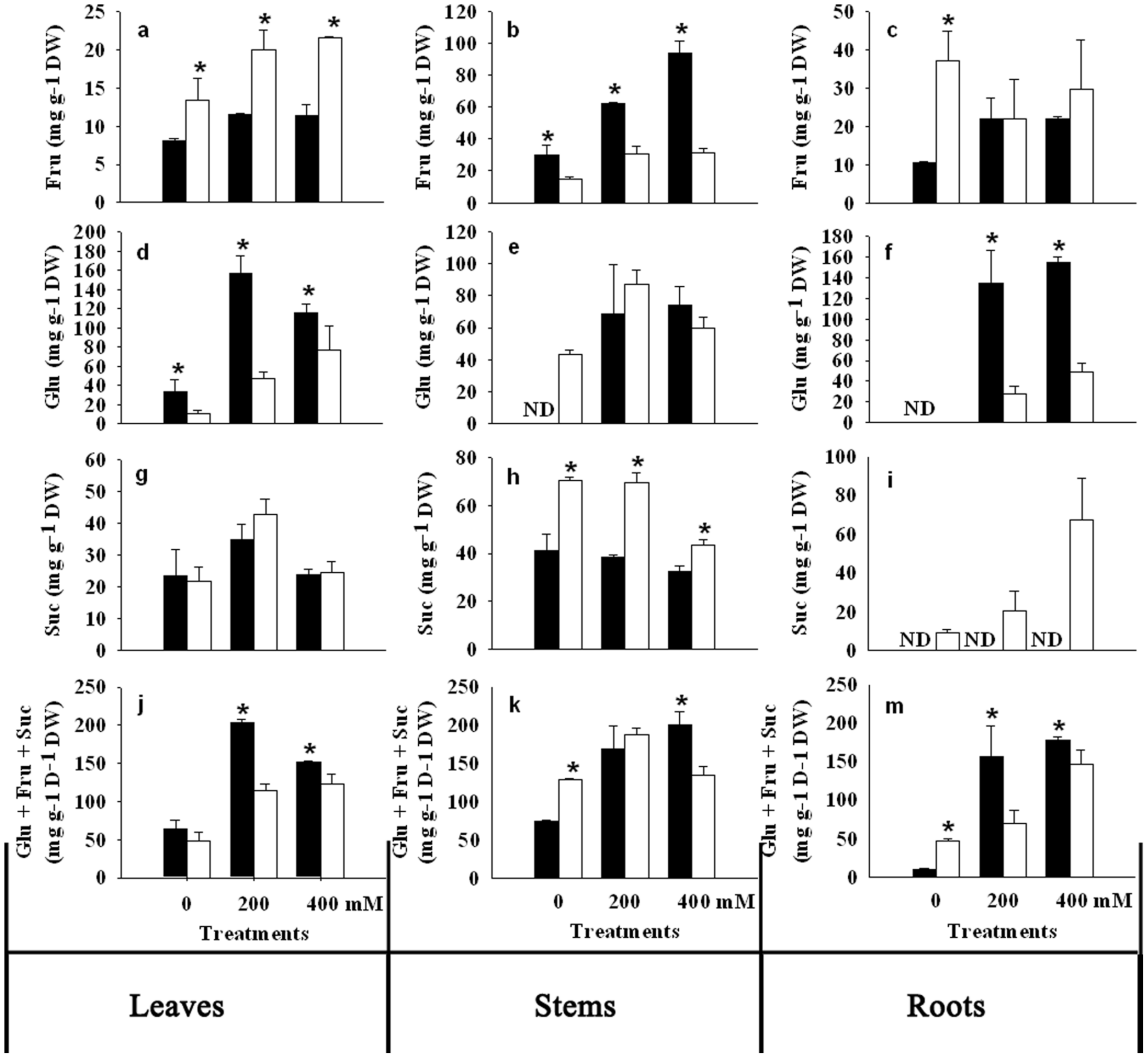
Effects of salt stress on the contents of fructose (Fru), glucose (Glu), sucrose (Suc), total soluble sugar (Glu+Fru+Suc) in leaves, stems and roots of two perennial ryegrass accessions (‘PI 538976’, black bar; ‘Overdrive’, white bar). Vertical bars represent means ± standard errors (n = 4) based on least significant difference (LSD) test (*P* ≤ 0.05). Asterisk symbols indicate significant differences between ‘PI 538976’ and ‘Overdrive’ (*P* ≤ 0.05).

Fructose (Fru) content was greater in root, stem and leaf of NaCl treated ‘PI 538976’, when compared to the control (Fig. 4a, b, c). Salinity stress led to an increase in stem and leaf Fru in ‘Overdrive’, but had no effects on root Fru. The unstressed roots and leaves of ‘Overdrive’ had a higher level of Fru than those of ‘PI 538976’ (Fig. 4a, c). However, there were no differences in Fru content in roots for both accessions under salinity stress (Fig. 4c). Leaf Fru content was higher in ‘Overdrive’ relative to ‘PI 538976’ (Fig. 4a). ‘PI 538976’ had a greater Fru content than ‘Overdrive’ under non-stressed and salt-stressed conditions in stems (Fig. 4b).

Salt stress significantly promoted glucose (Glu) accumulation in roots, stems and leaves of both grass accessions (Fig. 4d, e, f). ‘PI 538976’ was superior to ‘Overdrive’ in accumulating Glu in roots and leaves under non-salinity or salinity conditions (Fig. 4d, f). There was no difference in stems Glu content between ‘PI 538976’ and ‘Overdrive’ subjected to salinity during whole experimental period (Fig. 4e). Glu content was too low to be detected for roots and stems of non-stressed ‘PI 538976’ and for roots of non-stressed ‘Overdrive’.

The root sucrose (Suc) was not detected for ‘PI 538976’ regardless of salinity treatments. ‘Overdrive’ had greater stem and root Suc content than ‘PI538976’ when exposed to NaCl stress (Fig. 4 h, i). Leaf Suc content was greater in 200 mM NaCl regime versus other two treatments for both ‘PI 538976’and ‘Overdrive’. There was no difference in stem Suc contents of ‘PI 538976’ among three salinity treatments (Fig. 4 h). ‘Overdrive’ had a lower stem Suc content at 400 mM NaCl regime, when compared to the control (Fig. 4 h). ‘Overdrive’ had higher stem and root Suc content than ‘PI 538976’ regardless of NaCl treatments (Fig. 4 h, i). The two accessions had a similar level of leaf Suc in all salinity regimes.

Salinity stress increased soluble sugar (Glu+Fru+Suc) content in all tissues of both grass accessions (Fig. 4j, k, m). Under non salinity conditions, ‘PI 538976’ produced less soluble sugar in roots and stems than ‘Overdrive’. Under salinity stress (400 mM NaCl) conditions, ‘PI538976’ accumulated more soluble sugar in root and stem tissues than ‘Overdrive’ (Fig. 4k, m). ‘PI 538976’ accumulated more soluble sugar in leaves than ‘Overdrive’ under non-salinity or salinity conditions.

### Relationships among Photosynthetic Traits and Soluble Sugar Content

For ‘PI 538976’, Glu and GFS content in leaves, stems and roots negatively and significantly correlated with P_n_ under salt stress (*P*<0.01) and the same relationships in leaves and roots for ‘Overdrive’ (Table 2). Glu and GFS content in roots negatively correlated with all photosynthetic traits [P_n_ (*P*<0.05), g_s_ (*P*<0.01), C_i_ (*P*<0.05), F_v_/F_m_ (*P*<0.05) ] in both grass accessions under salt stress. Fru content in leaves and stems of both grass accessions negatively correlated with P_n_ (*P*<0.01) and g_s_ (*P*<0.01), except for P_n_ (*P* = −0.45) in ‘Overdrive’. In ‘PI 538976’ roots, Fru content was observed to negatively and significantly correlate with g_s_ (*P*<0.05) and C_i_ (*P*<0.05). Suc content in ‘Overdrive’ roots negatively correlated with all photosynthetic traits [P_n_ (*P*<0.05), g_s_ (*P*<0.05), C_i_ (*P*<0.05), F_v_/F_m_ (*P*<0.01) ]. Suc content in ‘Overdrive’ stems was observed to positively and significantly correlate with P_n_ (*P*<0.01), g_s_ (*P*<0.05) and F_v_/F_m_ (*P*<0.01). As a sink, Suc content in leaves had no significantly effects on all photosynthetic traits in both grass accessions.

**Table 2:** Correlations among photosynthesis traits, Fru, Glu, Suc and Glu+Fru+Suc (GFS) content in leaves, stems and roots of perennial ryegrass under salt stress.

Trait	Species	Leaves				Stems				Roots			
		Fru	Glu	Suc	GFS	Fru	Glu	Suc	GFS	Fru	Glu	Suc	GFS
CO_2_ assimilation (P_n_)	PI 538976	−0.94**	−0.83**	−0.53	−0.83**	−0.80**	−0.79**	0.47	−0.85**	−0.55	−0.79**	ND	−0.78**
	Overdrive	−0.45	−0.73**	−0.38	−0.79**	−0.84**	−0.38	0.69**	−0.24	0.30	−0.77**	−0.58*	−0.63*
Stomatal conductance (g_s_)	PI 538976	−0.98**	−0.95**	−0.44	−0.93**	−0.77**	−0.79**	0.39	−0.86**	−0.67*	−0.92**	ND	−0.91**
	Overdrive	−0.55*	−0.85**	−0.37	−0.89**	−0.83**	−0.57*	0.65*	−0.39	0.27	−0.89**	−0.62*	−0.72**
Internal CO_2_ concentration (C_i_)	PI 538976	−0.39	−0.55	−0.10	−0.51	0.00	−0.13	−0.13	−0.11	−0.65*	−0.57*	ND	−0.58*
	Overdrive	−0.16	−0.70**	0.09	−0.58*	−0.47	−0.33	0.50	−0.17	−0.22	−0.78**	−0.54*	−0.76**
Photochemical efficiency (F_v_/F_m_)	PI 538976	−0.48	−0.19	0.36	−0.14	−0.83**	−0.47	0.52	−0.64*	−0.46	−0.60*	ND	−0.59*
	Overdrive	−0.46	−0.77**	−0.12	−0.75**	−0.59*	−0.31	0.79**	−0.07	0.14	−0.85**	−0.76**	−0.82**

** and * indicate *P ≤* 0.01 and 0.05, respectively.

### Effects of Soluble Sugar Flux on Growth Traits and Salt Tolerance

**Figure 5:**
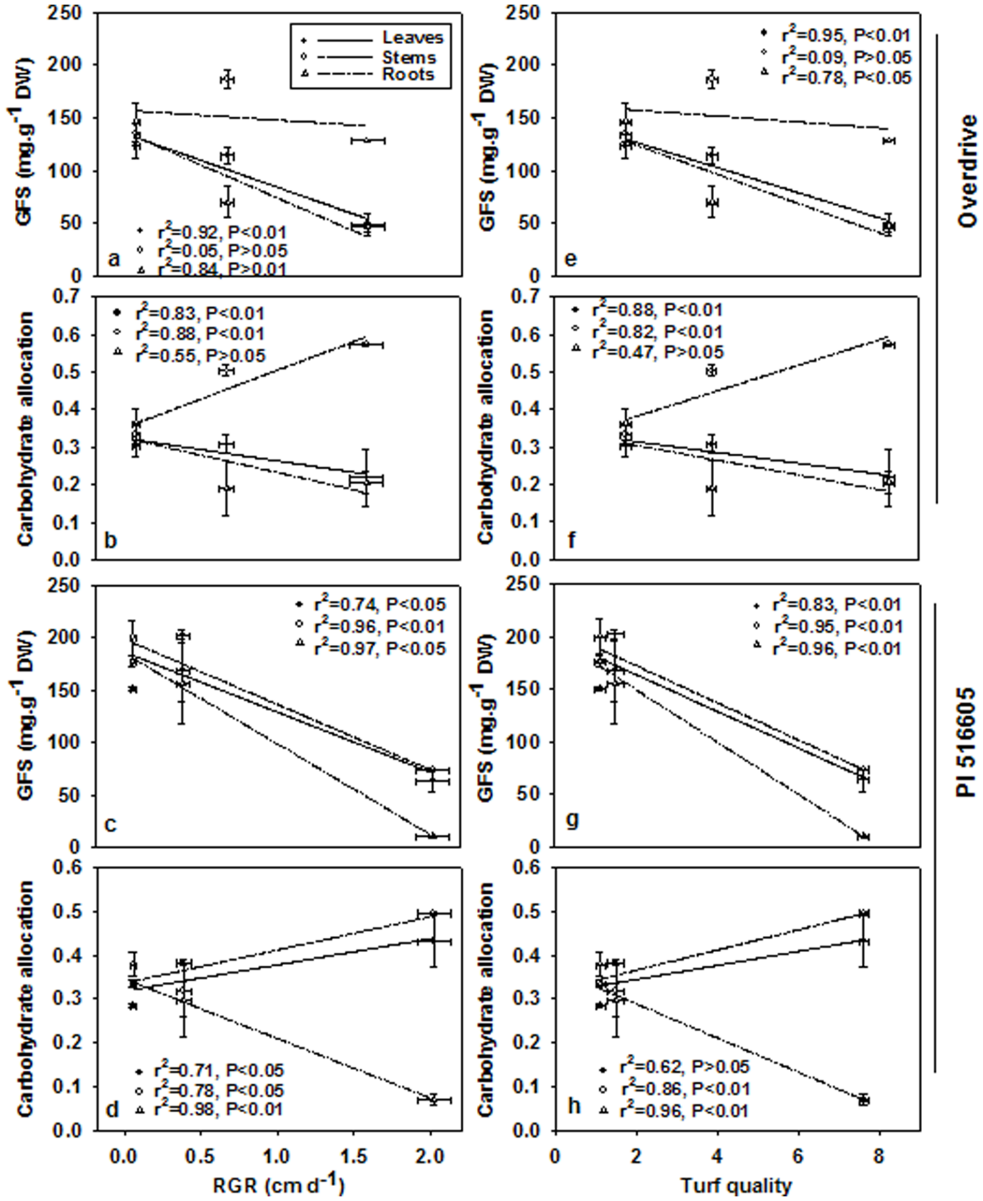
Correlations of GFS, carbohydrate allocation, RGR and turf quality in leaves, stems and roots of two perennial ryegrass accessions ‘PI 538976’ and ‘Overdrive’ in response to salt stress. Carbohydrate allocation was on behalf of the rate of GFS content in one tissue to total GFS content in plant.

GFS content in leaves negatively and significantly correlated with RGR in ‘Overdrive’ (*P*<0.01) and ‘PI 538976’ (*P*<0.05) under salt stress and the same relationship was observed in stems (*P*<0.01) and roots (*P*<0.05) for ‘PI 538976’ (Fig. 5a, c). GFS content in leaves, stems and roots of both grass accessions negatively and significantly correlated with turf quality, except for ‘Overdrive’ stems (Fig. 5e, g). As a soluble sugar flux index, carbohydrate allocation in stems positively and significantly correlated with RGR in both grass accessions (r^2^ = 0.88, *P*<0.01 for ‘Overdrive’; r^2^ = 0.78, *P*<0.05 for ‘PI 538976’) (Fig. 5b, d). For ‘Overdrive’, carbohydrate allocation negatively and significantly correlated with RGR (r^2^ = 0.83, *P*<0.01) in leaves (Fig. 5b). However, a positive and significant relationship between carbohydrate allocation and RGR was observed in ‘PI 538976’ leaves (r^2^ = 0.71, *P*<0.05) (Fig. 5d). Carbohydrate allocation positively correlated with turf quality in stems of both grass accessions (r^2^ = 0.82, *P*<0.01 for ‘Overdrive’; r^2^ = 0.86, *P*<0.01 for ‘PI 538976’) and negatively correlated with turf quality in ‘PI 538976’ roots (r^2^ = 0.96, *P*<0.01) (Fig. 5f, h). For ‘Overdrive’, carbohydrate allocation negatively and significantly correlated with turf quality (r^2^ = 0.88, *P*<0.01) in leaves (Fig. 5f). However, a positive correlation between carbohydrate allocation and turf quality was observed in ‘PI 538976’ leaves (r^2^ = 0.62, *P*>0.05) (Fig. 5 h).

### Gene Expression to NaCl in Roots, Stems and Leaves

**Figure 6:**
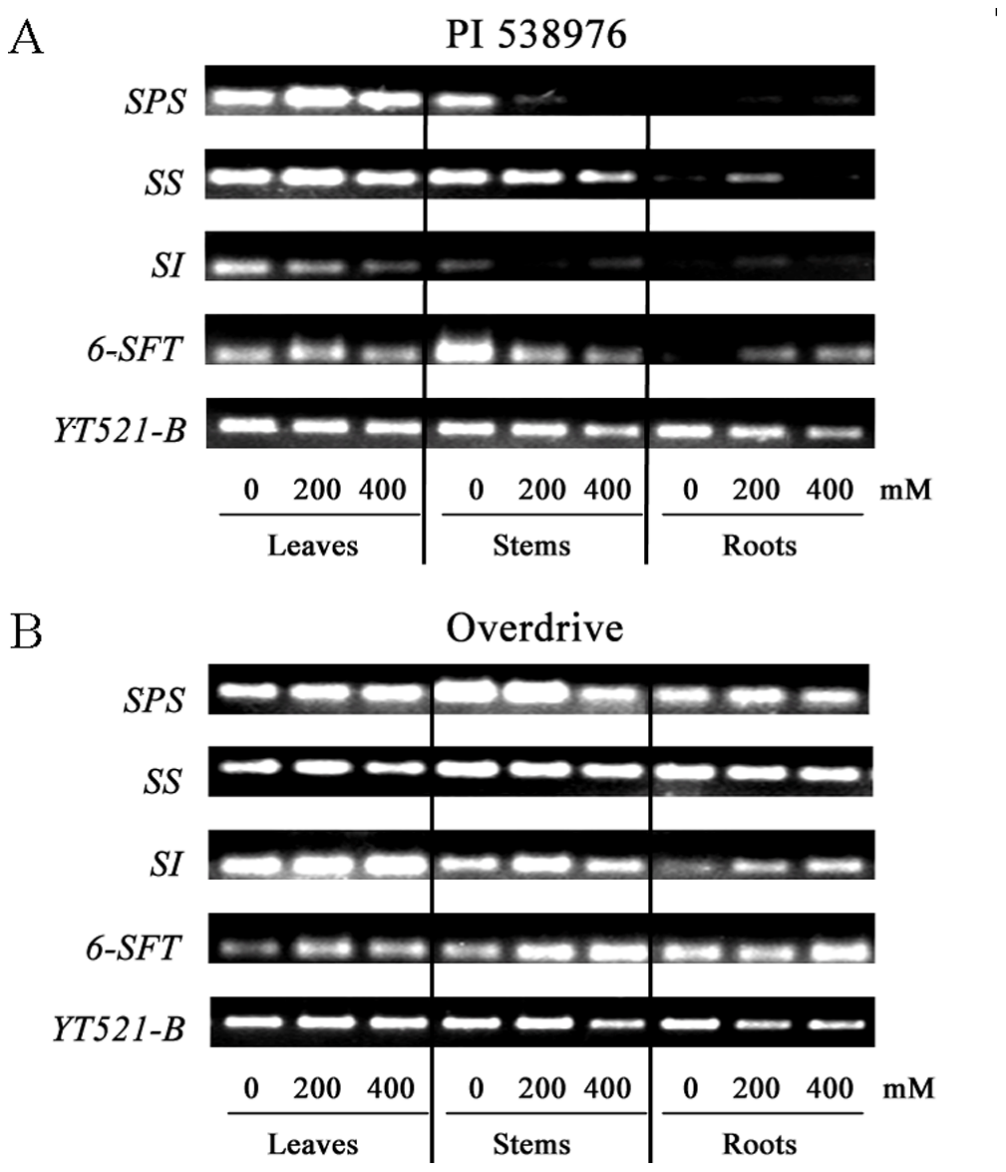
RT-PCR analyses of sucrose phosphate synthase (*SPS*), sucrose synthase (*SS*), cell wall sucrose invertase (*SI*), and sucrose: fructan 6-fructosyltransferase (*6-SFT*) genes in the leaves, stems and roots of ‘PI 538976’ (A) and ‘Overdrive’ (B) grown under normal (0 mM NaCl) or salt stress condition (treated with 200 or 400 mM NaCl for 5 days). *YT521-B* gene was used as the reference gene for cDNA normalization.

The expression of *SPS* in stems was lower for both accessions subjected to salinity. Both grass accessions exhibited a slight up-regulated expression of *SPS* in roots in response to salinity stress (Fig. 6). *SPS* expression was the highest for 200 mM NaCl-treated ‘PI 538976’ leaves (Fig. 6A). There was no difference in *SPS* expression between the control and salinity regimes for ‘Overdrive’ leaves (Fig. 6B). The highest root *SS* gene expression was found in ‘PI 538976’ treated with 200 mM NaCl. No difference *SS* expression was observed between the control and salinity regimes in all ‘Overdrive’ tissues. Stems and roots exhibited higher levels of *SS* transcripts relative to leaves in the ‘Overdrive’ at the same salinity level. In contrast, leaves exhibited higher level of *SS* expression relative to stems and roots in ‘PI 538976’. Higher levels of transcripts of *SPS* and *SS* gene in stem and root tissues were observed in ‘Overdrive’ relative to ‘PI 538976’ at the same salinity treatment. There was no difference in *SI* expression in ‘Overdrive’ leaves among all salinity treatments. Salt stress, however, inhibited *SI* expressions in ‘PI 538976’ leaves. *SI* expression was down-regulated in stems and up-regulated in ‘PI 538976’ roots subjected to 200 mM NaCl. ‘Overdrive’ exhibited an up-regulated *SI* expression in the 200 mM NaCl stressed roots. When subjected to the same level of salinity, *SI* expression of both grass accessions was the greatest for leaves, followed by stems and roots. The ‘Overdrive’ maintained a higher level of *SI* expression for all plant tissues compared with ‘PI 538976’ at the same level of salinity.

The expression of *6-SFT* was inhibited in the stems and induced in roots of ‘PI 538976’ when subjected to salinity stress. The expression of *6-SFT* in leaf tissue was up-regulated in ‘PI 538976’ when subjected to 200 mM NaCl. No significant difference was observed in leaf *6-SFT* between both grass accessions at the same level of salinity. Salt stress induced slightly expression of *6-SFT* in leaves and stems of ‘Overdrive’. A higher level of root *6-SFT* expression was observed in ‘Overdrive’ relative to ‘PI 538976’ at the same level of salinity. ‘Overdrive’ had a lower level of stem *6-SFT* expression in the control and a higher level of *6-SFT* expression under the salinity regimes than ‘PI 538976’.

## Discussion

The present study indicated that salt stress caused toxicity to perennial ryegrass. In response to salt stress, RGR decreased significantly for two perennial ryegrass accessions. The lower relative shoot growth rate of salinity-stressed plants was consistent with the previous results in mangrove (*Rhizophora mangle*) [40], [41], maize (*Zea mays*) [7], [42]. The decreased growth rate of salinity-stressed shoots may be resulted from decrease in division and expansion of plant cell [43]–[45] and higher apoplastic level of ions in the cell or inside-negative electrochemical gradient [46], [47]. The unbalance of photosynthesis and respiration also contributed to the decrease in growth rate [48], [49]. No significant difference in RGR were found between ‘PI 538976’ and ‘Overdrive’ in control regimes. However, salt tolerant ‘Overdrive’ exhibited descent RGR at less extent and a little greater growth rate than salt sensitive ‘PI 538976’. The results of this study along with previous studies suggested that reduction in growth rate may be related with the sensitivity to salinity in different plants [40], [50].

Normalized relative transpiration has been considered as an indicator of toxicity effects in the salinity stressed plants [34], [51], [52]. Salinity reduced NRT of the two accessions regardless of salinity regimes. Similar results have been obtained from citrus (*Citrus reticulata* Banco) [53], [54], rice [55], willow (*Salix* spp.) [51]. The reduction in transpiration may be contributed by injuries or decreases of hydraulic conductance in roots [56], the decrease of water potential (*ψ*_1_) and stomatal closure in leaves [54]. The present study indicated that a greater reduction in NRT was observed at 2 DAT in salt-sensitive ‘PI 538976’ relative to ‘Overdrive’ subjected to 200 mM NaCl. Furthermore, ‘Overdrive’ maintained better turf quality than ‘PI 538976’ at 200 mM NaCl. This suggests that salt sensitive accession may reduce NRT at greater extent than salt tolerant accession in response to salinity stress.

Previous investigations demonstrated that salt stress resulted in an inhibition to PSII activity [10], [57]–[59]. However, Morales et al. [60] have reported that salinity did not affect PSII of most barley leaves. Our results showed that F_v_/F_m_ decreased significantly for the two accessions at 400 mM NaCl treatment relative to the control. NaCl at 200 mM had no effects on the F_v_/F_m_ of salt-sensitive ‘PI 538976’. ‘Overdrive’ had a higher F_v_/F_m_ than ‘PI 538976’, when exposed to 400 mM NaCl. These results indicated that only severe salinity stress caused reduction in F_v_/F_m_ and ‘PI 538976’ was more sensitive to severe salinity stress than in ‘Overdrive’. This suggests that severe salinity may directly damage PSII rather than changes electron transport activity [61]–[63].

The present study indicated that moderate salinity (200 mM NaCl) resulted in dramatic decline in net P_n_, C_i_, g_s_ and NRT for both accessions. The results suggested that the earlier photoinhibition of perennial ryegrass may be mainly caused by the diffusion limitations through the stomata and the mesophyll. This is in agreement with the results obtained in bean [8], bean and cotton [5] and cotton [6], and olive trees (*Olea europaea* L.) [64]. P_n_ remained unchanged for ‘Overdrive’ as NaCl concentration increased from 200 mM to 400 mM. For ‘PI 538976’, increase in C_i_ and low levels of P_n_, g_s_ and NRT of suggested that the lower Pn may be mainly resulted from non-stomatal limitation for the salt sensitive plants. Non-stomatal limitation has been considered as the major cause for the alterations of photosynthetic metabolism at high salinity levels.

Soluble sugar (i.e. sucrose, glucose and fructose) is the major existence form of products of photosynthesis in C_3_ grasses, in which the sugar is translocated from source tissue to the sink tissues such as roots and stems [18], [65], [66]. The results of this study indicated that, plant sink tissues (i.e. roots, stems and leaves) accumulated more soluble sugars (Fig. 4) despite decreased P_n_ (Fig. 3). Salt-sensitive accession accumulated greater amount of soluble sugars in the sink tissues than salt-tolerant accession in response to salt stress. These results suggested that soluble sugar play a critical role in regulating the CO_2_ assimilation for plant adaptation to salt stress [43], [67]. This inference was also supported by the results that leaf soluble sugar content in both grass accessions was generally negatively correlated with F_v_/F_m_, C_i_ and g_s_ (Table 2). Soluble sugar in the major sink tissue (roots, stems) of both grass accessions was generally negatively correlated with CO_2_ assimilation rate. This supported the hypothesis that feedback repression from source–sink regulation by carbohydrate may fine tune the rate of CO_2_ assimilation [26], [67], [68]. Furthermore, GFS content in all tissues (leaves, stems and roots) of both grass accessions negatively and significantly correlated with turf quality, except for ‘Overdrive’ stems (Fig. 5e, g). GFS content in leaves negatively and significantly correlated with RGR in ‘Overdrive’ (*P*<0.01) and ‘PI 538976’ (*P*<0.05) under salt stress and their relationship was observed for stems (*P*<0.01) and roots (*P*<0.05) in ‘PI 538976’ (Fig. 5a, c). These indicated that soluble sugar excessively accumulated in source–sink tissues partially inhibited the plant growth. In addition, in leaves, carbohydrate allocation negatively and significantly correlated with RGR (r^2^ = 0.83, *P*<0.01) and turf quality (r^2^ = 0.88, *P*<0.01) in salt-tolerant ‘Overdrive’, however, the opposite trend for salt-sensitive ‘PI 538976’ (r^2^ = 0.71, *P*<0.05 for RGR; r^2^ = 0.62, *P*>0.05 for turf quality). These indicated that plant salt tolerance may be changed with the transformation of soluble sugar flux in different sink tissues (leaf, stem and root).

Sucrose is the major form of carbohydrates for translocation [69]. Previous studies indicated that excess accumulation of sucrose can feed forward to activate signal transduction pathways in sink processes and feed back to down-regulated photosynthesis [70]–[73]. If the decline in photosynthesis was induced by the feedback regulation due to excess accumulated sucrose, it could be hypothesized that excess sucrose accumulated in sink tissue when the photosynthesis declined. Salinity did not affect leaf Suc content for both accessions subjected to 400 mM NaCl in spite of decrease in P_n_. Root Suc content was detected in ‘Overdrive’ as NaCl concentration increased. In ‘PI 538976’ root, Suc content showed large negative correlations with photosynthetic traits (*P*<0.05 for P_n_, g_s_, C_i_; *P*<0.01 for F_v_/F_m_; Table 2). This suggests that the effects of Suc negative feedback regulation in signal transduction pathways induced by salt stress in root cell.

Hexoses (i.e. Fru and Glu) are important signal molecules in source–sink regulation, which can modulate gene expression encoding photosynthetic proteins [71], [73], [74]. Our results showed that salt stress significantly promoted Glu accumulation in roots, stems and leaves of both grass accessions. However, salt-sensitive ‘PI 538976’ accumulated higher Glu content in salinity-stressed roots and leaves than ‘Overdrive’. Fru also accumulated in stems and leaves of both accessions under salt stress. As accumulation of hexoses in sink tissues increased, the leaf photosynthetic rate decreased. This is in agreement with the result with citrus leaves in response to boron stress [75]. Furtherly, Glu content in roots negatively correlated with all photosynthetic traits [P_n_ (*P*<0.05), g_s_ (*P*<0.01), C_i_ (*P*<0.05), F_v_/F_m_ (*P*<0.05) ] in both grass accessions under salt stress. And Fru content in leaves and stems of both grass accessions negatively correlated with P_n_ (*P*<0.01) and g_s_ (*P*<0.01), except for P_n_ (*P* = −0.45) in ‘Overdrive’. These results indicate that a negative feedback repression of photosynthesis may take place through accumulation of hexoses in the sink tissues under salinity conditions in perennial ryegrass.

In plant cells, the mutual transformation among sucrose, glucose and fructose proceed according to the readily reversible reaction: sucrose ↔ glucose+fructose, [76]. The regulation of the metabolism reaction might be attributed to up and down expression of the candidate gene [29]. The expression of *SPS* and *SS* was decreased with the increasing salinity concentration in ‘PI 538976’ stems, while *SPS* expression was also lower in ‘Overdrive’ stems. The *SI* expression was induced by salt stress (200 mM NaCl) in ‘Overdrive’ stems. If expression of both *SPS* and *SS*, which encoding enzymes for biosynthesis of Suc, was down-regulated and further the expression of *SI* encoding enzymes to converted Suc was inducted in salinity-stressed stems, it can be hypothesized that stems Suc content decreased in the same salinity-stressed tissues. The results of this study indicated that stem Suc content decreased and both Fru and Glu content increased in both grass accessions. This suggests that gene regulation may have effects at transcription level or soluble sugars feeding may effectively induce or repress gene expression in stems of perennial ryegrass [77], [78].

In the roots of both grass accessions, the increased *SI* expression was not concomitant with a similar decrease in expression of both *SPS* and *SS* gene. In salt sensitive ‘PI 538976’ roots, there was a dramatic increase of *SPS* and *SI* gene expression with the increase of NaCl concentration and *SS* gene was significantly induced at 200 mM NaCl. Similarly, *SI* and *SPS* gene expression in root tissue increased in ‘Overdrive’ exposed to 200 mM NaCl. We further found that salinity-stressed roots of ‘Overdrive’ had more Suc and Glu. This suggests the induction of all *SPS*, *SS* and *SI* genes may play a key role in maintaining the balance of Suc metabolism in systemic acquired resistance in response to salt stress [27], [79].

The reaction (sucrose→ fructan+ glucose) is catalyzed by sucrose: fructan 6-fructosyltransferase in cool-season grasses cells [78], [80]. Our results indicated that stem Suc content decreased, but Glu content increased in NaCl treated ‘Overdrive’. Here we also found that salt stress induced slightly the expression of *6-SFT* in all plant tissues of tolerant ‘Overdrive’. In salinity-stressed roots of sensitive ‘PI 538976’, both *6-SFT* expression and Glu content increased. These results indicated that *6-SFT* may play an important role in converting sucrose into fructan and glucose [81]. The levels of *SPS*, *SS*, *SI*, *6-SFT* expression in all tissues were higher in salt-tolerant ‘Overdrive’ than that in salt-sensitive ‘PI 538976’. This suggests that tolerance to salt stress was at least in part associated with up-regulated expression of carbohydrate metabolism gene.

### Conclusions

In conclusion, an accumulation of free hexoses in roots, stems and leaves can repress photosynthesis in salinity-stress perennial ryegrass. There is evidence here that high carbohydrate allocation in stems and low carbohydrate allocation in leaves may play a key role in improving salt tolerance of perennial ryegrass. The grass tolerance to salt stress was in part associated with up-regulated expression of carbohydrate metabolism gene (*SPS*, *SS*, *SI*, *6-SFT*). Soluble sugars accumulation effectively induced or repressed *SPS*, *SS*, *SI* gene expression in stems of perennial ryegrass. The present work also showed that maintenance of higher CO_2_ assimilation capacity (higher P_n_, F_v_/F_m_ and lower g_s_, C_i_ at 400 mM NaCl) under salt stress in salt-tolerant relative to salt-sensitive perennial ryegrass accessions attributed to higher RGR, NRT, turf quality, expression of soluble sugars metabolism gene (*SPS*, *SS*, *SI*, *6-SFT*) and lower levels of accumulation in soluble sugars in sink tissues. The carbohydrate allocation and source–sink regulation induced by sugars can help us better understand salt stress tolerance and breeding salt-tolerant grasses.
